# Role of sleep problem on suicidal behaviour and non-suicidal self-injury among adolescents in Pokhara, Nepal

**DOI:** 10.1371/journal.pone.0305221

**Published:** 2024-09-25

**Authors:** Seema Thapa, Dipendra Kumar Yadav

**Affiliations:** School of Health and Allied Sciences, Pokhara University, Pokhara, Nepal; Research, Training and Management International, BANGLADESH

## Abstract

**Introduction:**

Sleep is a fundamental human requirement, considered one of the major contributing factors to physical and mental health, especially among adolescents. Poor quality sleep has many potential consequences including non-suicidal self-injury (NSSI), suicidal thoughts or behaviour and complete suicide. The NSSI and suicidal behaviour are increasing in Nepal. Thus, this study aimed to assess the role of sleep problem on suicidal behaviour and non-suicidal self-injury among adolescents in Pokhara metropolitan, Nepal.

**Methods:**

A cross-sectional analytical study was conducted among 673 adolescents using a multistage cluster sampling technique from private and public schools in Pokhara Metropolitan. Self-administered questionnaire was used for data collection. Collected data was entered and managed in EpiData (version 3.1) and analysed in IBM SPSS (version 23). Binary logistic regression was used to identify the association of sleep problem with NSSI and suicidal behaviour.

**Results:**

The study found that sleep problem (65.2%, 439), suicidal behaviour (18.6%, 125) and NSSI (57.9%, 390) were prevalent among adolescents in study setting. The presence of sleep problem significantly influenced the suicidal behavior (AOR = 3.88, 95% CI = 2.27–6.63) alongside the sex of student (AOR = 1.96, 95% CI = 1.28–3.00), adolescents from family having monthly income less than NPR 40,000 (AOR = 1.97 95% CI = 1.16–3.35) and private schools students (AOR = 2.99, 95% CI = 1.84–4.86). Likewise, sleep problem was also associated with non-suicidal self-injury (AOR = 3.24, 95% CI = 2.26–4.65), in addition to attending private school (AOR = 2.52, 95% CI = 1.71–3.72).

**Conclusion:**

This study concludes that sleep problem is prevalent among the adolescents and increase the risk of NSSI and suicidal behaviour. Therefore, parents and teachers need to assess their conditions and help them maintain sound sleep. Additionally, suicide prevention strategies need to be adopted to mitigate further risk.

## Introduction

Sleep problems among adolescents are a highly prevalent issue worldwide, with prevalence rate ranging from 1.6% to 56% [1–3]. Sleep problem is the condition of having difficulty in initiating sleep, difficulty in maintaining sleep and early morning awakening [4]. The recommended amount of sleep for adolescents who are 13 to 18 years is 8 to 10 hours [5].

Numerous factors contribute to sleep quality among undergraduate students, with significant determinants including gender, smoking habits, and physical activity [6]. Furthermore, among secondary school students, poor sleep is found to be correlated with variables such as age, public education, as well as symptoms of depression and anxiety [7]. Moreover, a heightened prevalence of sleep difficulties is observed in children from families characterized by lower socioeconomic status. Additionally, maternal educational attainment was associated with diminished duration of time spent in bed [8].

Suicidal behaviours are classified mainly into four categories: suicide ideation, which refers to thoughts of engaging in behaviour intended to end one’s life; suicide plan, which refers to the formulation of a specific method through which one intends to die; suicide threat that refers to any verbal or non-verbal action intended to communicate that suicidal behavior might occur in near future and suicide attempt, which refers to engagement in potentially self-injurious behaviour in which there is at least some intent to die [9]. Globally, suicidal ideation ranges from 6.64% to 17.53%, suicidal plan from 7.89% to 15.29% and suicidal attempt from 6.44% to 15.99% [10]. While in Nepal, prevalence of suicidal behaviour is (suicidal ideation = 13.7%, suicidal attempt = 10.0% and suicidal plan = 14.0%) [11].

Suicidal ideation and attempts significantly increases in later teenage groups [12,13]. Female adolescents, academic stress in private schools, adolescents boarding at school and those with poor school performances are more likely to engage in suicidal behaviour [13–15]. Adolescents who have short sleep duration and economic difficulty in families are prone to suicidal ideation [16].

Non suicidal self-injury is an act of deliberately and directly destruction of owns body tissue without an intent to die which mainly characterized by biting self, cutting/carving skin, self-hitting body parts, burning skin scratching, banging, interfering with wound healing [17,18].

The rates of lifetime and past 12-month prevalence of non-suicidal self-injury varied across different regions in the world ranging from 18.4% to 30.9% [19]. In Nepal, about 45% adolescents had a history of non-suicidal self-injury in past 1 year [18].

NSSI is often multifactorial where female adolescents and students from private school reported more NSSI [18]. A meta-analysis by Luca et.al reported higher NSSI for younger adolescents [20]. Ethnic minority groups (Muslims born in Israel and immigrants from the former Soviet Union) indicated that they engaged in NSSI [21]. Likewise, adolescents from low socio economic status category and with sleep disturbances were more likely to self- harm [22,23].

Adolescents with sleep disturbances have twice the risk of suicidal ideation, plans and attempts compared to those without sleep disturbances [24–29]. These findings were also supported by longitudinal studies [30,31]. Similarly, non-suicidal self-injury was associated with sleep problems like short sleep duration, sleep disturbances, and poor sleep quality [32]. Likewise, studies have reported that poor sleep quality was significantly and independently associated with non-suicidal self-injury [13,24–26]. These findings were also supported by a longitudinal study conducted among Swedish adolescents [33].

Mental health issues are rarely addressed in schools and within families. The failure to recognize and address mental health problems in children and adolescents is a serious public health issue with important consequences on the achievement of basic development goals in low and middle-income countries like Nepal [34,35]. In Nepal, the prevalence of sleep problems among adolescents ranges from 24.4% to 39.1%, displaying an increasing trend in different semi urban setting [36–38]. Internet addiction emerges as a significant contributing factor to sleep disturbances in this place [36,37]. Concurrently, suicidal behavior is a pressing concern, with suicidal ideation ranging from 10.4% to 13.7%, suicidal plans from 7.9% to 14%, and suicidal attempts from 4.5% to 10.33% [11,18,39]. Notably, non-suicidal self-injury in Nepal is reported at 45% in semi urban setting, aligning with the global trend of 7.5% to 46.5%, and displaying an increasing pattern among adolescents [18,40]. Females, alongside factors such as anxiety, loneliness, and depression, are identified as common contributors to suicidal behavior and non-suicidal self-injury in the Nepalese adolescent population [18,39]. Despite the high prevalence of sleep problems, suicidal behavior and non-suicidal self-injury, the specific relationship between sleep problems and these adverse outcomes remains unknown in the Nepalese context. Evidences from studies conducted outside the country has indicated an association of sleep problem with suicidal behaviour and NSSI [25,32]. Therefore, this study aimed to assess the role of sleep problem on suicidal behaviour and non-suicidal self-injury among adolescents in Pokhara metropolitan.

## Methods and materials

### Study design and setting

School based cross-sectional analytical study design was adopted to conduct the study in Pokhara Metropolitan city. It is the Nepal’s largest Metropolitan city by area occupying 464.24 km^2^ with 33 wards. The total population of Pokhara Metropolitan is 513,504 [41].

### Study participants

The study participants were individual school students, ranging from class 9 to class 12, adolescents aged 13 to 19 years, selected from the schools of Pokhara Metropolitan City. Top of FormLikewise, students on medication for severe types of sleep problems, suicidal behaviour and non-suicidal self-injury were not included in the study.

### Sample size and sampling technique

There were total 240 schools in Pokhara Metropolitan, 140 private and 100 public schools. Among which there were (74 public and 140 private) secondary schools as per Education section, Pokhara Metropolitan. Sample size was calculated using sample size calculation option available in OpenEpi website considering the proportion of 44.8% non-suicidal self-injury adolescent student [18].

Sample size *n* = [DEFF*Np (1-p)]/ [(d^2^/Z^2^_1-α/2_*(N-1)+p*(1-p)]

Where,

Z = value of standard normal distribution in 1.96 level of significant with 95% confidence level

p = 44.8% (prevalence of non-suicidal self-injury among adolescents)

q = (1- p) = (1–0.448) = 0.552

d = desirable error 0.05 (5% margin of error)

For infinite population (population more than 10,000) N = 1000000

DEFF = Degree of freedom = 1.5 (default)

Now, putting the values and adding 10% non-response rate the final sample size obtained was n = 634

A total of 673 students were recruited through multistage cluster sampling.

PPS followed for sample size allocation.

**Fig 1:**
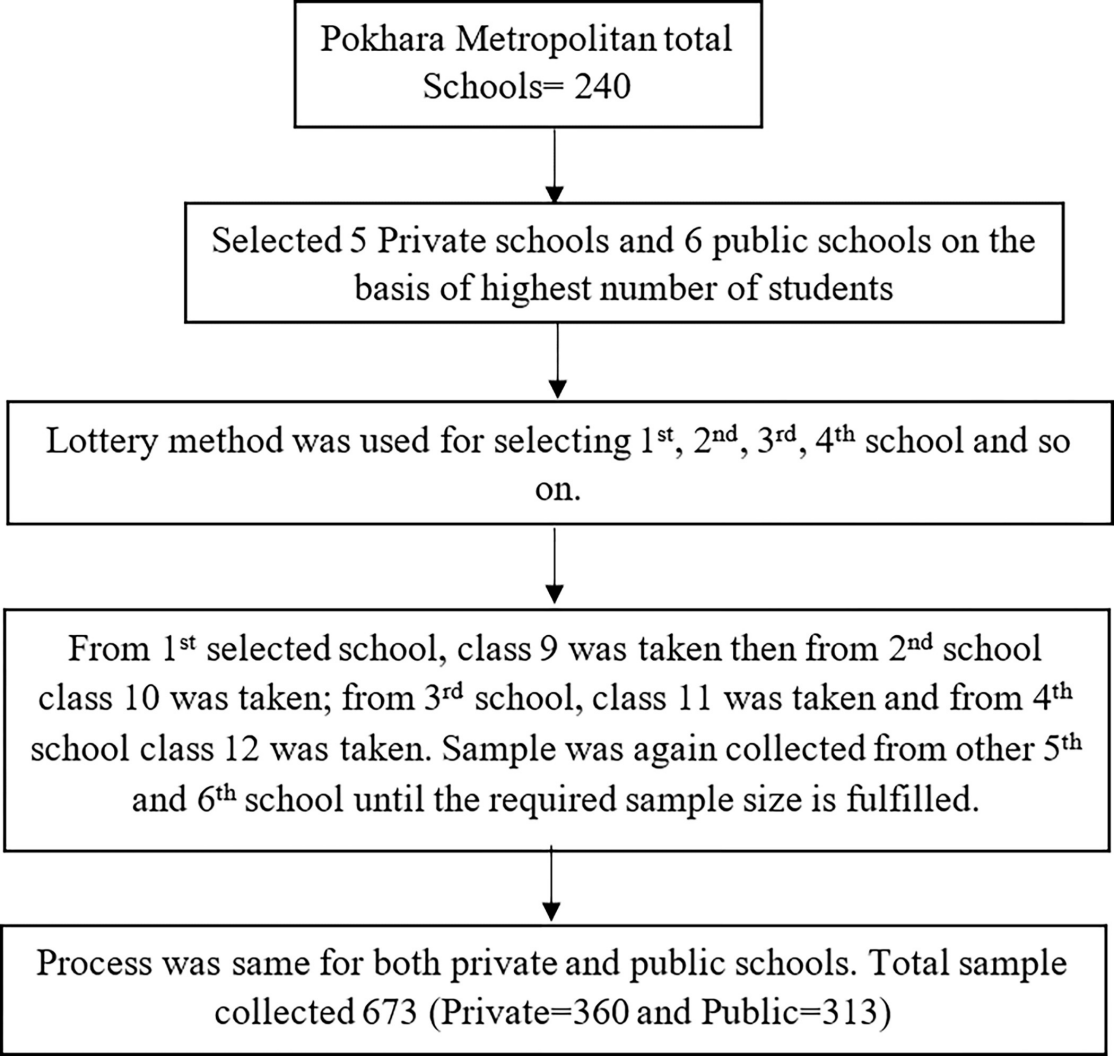
Flow diagram of study participants’ selection.

Firstly, list of the public and private schools and number of students of each class was obtained from Education section, Pokhara Metropolitan. Secondly, six schools from private and public sector were selected on the basis of highest number of students. The number of sample from each school was estimated based on Population Proportionate to Size (PPS). Then lottery method was used for selecting 1^st^, 2^nd^, 3^rd^, 4^th^ school and so on. From 1^st^ selected school, class 9 was taken then from 2^nd^ school class 10 was taken; from 3^rd^ school, class 11 was taken and from 4^th^ school class 12 was taken. Sample was again collected from other 5^th^ and 6^th^ school until the required sample size is fulfilled. This process was same for both private and public schools. The total sample size collected was 673 where 360 samples were collected from private schools and 313 samples were collected from public schools (Fig 1).

### Data collection

A semi-structured self-administered questionnaire was used for data collection. The tool composed of four sections and socio-demographic section was prepared through literature review and revised after pre testing. Data collected during pretesting were not included in the final analysis. Data collection was done from 26^th^ June 2023 to 27^th^ July 2023.Data collection was facilitated by first author herself in Nepali language. During the data collection process, students were initially instructed to obtain assent from their parents. The following day, students who secured both parental assent and consent were provided with an explanation of the questionnaires in the presence of the researcher herself. The researcher instructed the students to seek clarification by asking questions if they encountered any confusion. It was clarified that participation in the study was voluntary, with the option to withdraw if they chose not to continue. Thirty students did not obtain parental assent; consequently, data were not collected from those students.

### Measures

#### Socio demographic information

It included participants’ age, sex, ethnicity, religion, family income, mother and father education, mother and father occupation and type of school (Public, Private).

#### Sleep problem

In this study, student version of the Child and Adolescents Sleep Checklist (CASC-s) was used which has previously been employed in Nepal [38,42]. Permission was taken from author to use Nepali version of their CASC-s tool in the study to assess adolescents’ sleeping patterns and disturbances. The instrument aims to assess the sleep patterns of pre-schoolers and school children, including high school students. The CASC-Student version comprises 24 questions addressing sleeping problems. Responses to these 24 items were recorded on a four-point Likert scale, with 0 indicating never, 1 indicating occasionally (1day or less per week), 2 indicating sometimes (2 to 4 days per week), and 3 indicating always (5 to 7 days per week). The total score of the 24-item sleep disturbance test ranges from 0 to 72. A CASC score of 18 or higher is indicative of a sleep problem in children [42]. Moreover, the CASC score is sub-divided into the following four categories: "bedtime problem" (Q1-Q6), "sleep breathing and unstable sleep" (Q7-Q12), "parasomnia," "sleep movement," (Q13—Q18) and "daytime problems" (Q19—Q24) [42]. The tool’s Cronbach’s alpha in this study was 0.805. Similarly, obstructive sleep apnea (OSA) symptoms which is characterized by partial or complete obstruction of upper airways, was assessed by two-items related to individual snoring behavior and sleepiness feeling. Respondents was categorized as having OSA symptoms if they answered "true" or "mostly true" to the statements "I snore (or someone else claims I snore)" and "feeling sleepy at least three days per week". This particular definition of symptoms related to obstructive sleep apnea has been employed in past epidemiological studies [43,44].

#### Non suicidal self–injury

The Functional Assessment of Self-Mutilation (FASM) tool was used to measure non suicidal self-injury [45]. Twelve different non suicidal self -injury behaviours are listed in the FASM, with the following behaviours being classified as "minor non suicidal self-injury ": hitting oneself, pulling one’s hair, biting oneself, putting objects under one’s nails or skin, picking at a wound, and picking areas to draw blood; "moderate/severe non suicidal self-injury ": cutting/carving, burning, self-tattooing, scraping, and "erasing" (i.e. using an eraser to rub skin to the point of burning and bleeding) skin [17].

Among teenage samples, the FASM was shown to have acceptable psychometric properties, generating adequate internal consistency ranging from 0.62 to 0.85 for each subscale of non-suicidal self-injury functions [46]. In this study, Cronbach’s alpha value for FASM tool was 0.768.

#### Suicidal behaviour

Using the Suicidal Behaviours Questionnaire-Revised (SBQ-R), suicidal behaviour was assessed [47]. SBQ-R tool assess four behaviours such as suicidal ideation, suicidal plan, suicidal attempt and suicidal threat. In this study all the four types of behaviour were assessed in order to determine student’s suicidal behaviour. Suicidal behaviour items were assessed using a Likert scale, where 5 to 7 response options per item were provided. Then scores from each item were summed to generate total score (range 3–18). Higher scores indicate higher suicidality levels. Also, the non-clinical high school adolescents sample’s cut-off score is7. (i.e., a score of 7 or above is classified as suicidal) [47]. In the Osman et al. study, the internal consistency coefficients of the SBQ-R ranged from 0.76 to 0.88 [47]. In this study, Chronbach’s alpha value for SBQ-R tool was 0.745.

#### Ethical statement

Ethical approval for the study was obtained from Institutional Review Committee of Pokhara University (reference number: 139-079/080). Permission was obtained from Pokhara municipality and selected schools. Confidentiality of the information was maintained, and information was used only for research and study purpose. Participants was asked to collect the signature on the assent form from their parents after explaining about the study details. In addition, written consent was also taken from participants. Then the next day, data were collected from students. Each participants was given right to withdraw from the study at any time during the data collection as per their wish and interest. All participants was informed that there was no monetary benefit nor any risk involved for the participants under this study. Further, adolescents who had sleep problem, non-suicidal self- injury and suicidal behaviour were counselled and suggested to visit health facilities for further diagnosis and treatment.

### Data management and Statistical analyses

The collected data was entered and managed in EpiData version 3.1 and exported to IBM SPSS version 23 for data analysis. Descriptive analysis such as frequency, percentage, median and IQR was measured based on data distribution. For inferential analysis, binary logistic regression was applied to identify the role of sleep problem on suicidal behaviour and non-suicidal self-injury. Variables with p-value<0.2 in unadjusted models included in the multivariate binary logistic regression model at 95% Confidence Interval (CI) for examining independent association between explanatory variables and dependent variables. However, in the multivariate binary logistic regression, only variable with p-value< 0.05 considered significant.

## Results

### Background characteristics

Table 1 shows that out of 673 students, around two third (64.8%) of them were middle adolescents. The participants median age was 16 (IQR = 2, Min-Max = 13–19) and median family income was NRS50000 ($381.0821 USD) per month. Regarding respondents mother and father educational status, more than half (57.2% and 57.6%) had completed their secondary level education respectively. Regarding participants mother occupation, more than one in four mothers were housewife (27.8%). Likewise, more than one quarter of participant’s father were involved in business (28.5%) and service (26.5%). Almost equal percent of students were from private (53.5%) and public schools (46.5%).

**Table 1:** Socio-demographic characteristics of adolescents in Pokhara Metropolitan.

Characteristics (n = 673)	Frequency	Percentage
**Age**		
Early adolescents (13–14)	126	18.7
Middle adolescents (15–17)	436	64.8
Late adolescents (18 and above)	111	16.5
(Median = 16, IQR = 2, Minimum = 13, Maximum = 19)		
**Sex**		
Male	367	54.5
Female	306	45.5
**Ethnicity**		
Brahmin	236	35.1
Janajati	214	31.8
Chhetri	122	18.1
Dalit	89	13.2

Madhesi	10	1.5
Muslim	2	0.3
**Mother education (n = 670)**		
Illiterate	61	9.1
Primary	136	20.3
Secondary	383	57.2
Bachelor and above	90	13.4
**Father education (n = 667)**		
Illiterate	31	4.6
Primary	123	18.4
Secondary	384	57.6
Bachelor and above	129	19.3
**Mother occupation (n = 670)**		
Housewife	186	27.8
Business	167	24.9
Farmer	128	19.1
Service	94	14.0
Unemployed	55	8.2
Foreign job	24	3.6
Daily wage labor	16	2.4
**Father occupation**		
Business	190	28.5
Service	177	26.5
Foreign job	148	22.2
Farmer	93	13.9
Daily wage labor	42	6.3
**Monthly family income**		
NRS <40000 ($304.8657 USD)	254	37.7
NRS 40001–50000 ($304.8733–381.0821 USD)	202	30.0
NRS 50001 and above ($381.0897 USD and above)	217	32.2
(Median = 50000 IQR = 50000, Minimum = 1000,Max = 500000)		
**Type of school**		
Private	360	53.5
Public	313	46.5
$1 USD = NRS131.2053		

### Sleep problem, suicidal behaviour and NSSI prevalence rates

Two third (65.2%) of adolescents had sleep problem whereas one third (34.8%) of them had no any sleep problem.

Table 2 shows that the prevalence for lifetime suicidal ideation, suicidal plan and suicidal attempts were 18.4%, 6.5% and 4.2% respectively. Regarding frequency of suicidal ideation in the past 1 year, one in ten (11.3%) had for once, less than one in ten (7%, 2.7% and 1.8%) had for twice, 3–4 times and 5 or more times respectively. Less than one tenth (6.9%) had ever told someone that they were going to commit suicide, or that they might do it. About one tenth (8.6%) of adolescents will attempt suicide someday.

**Table 2:** Extent of suicidal behaviour among adolescents in Pokhara, Nepal.

Variables (n = 673)	Category	Frequency	Percentage
Life time suicidal ideation, intent and/or attempts	Never	477	70.9
	Suicidal ideation	124	18.4
	Suicidal plan	44	6.5
	Suicidal attempts	28	4.2
Frequency of suicidal ideation in the past 1 year	Never	520	77.3
	Rarely (1 time)	76	11.3
	Sometimes (2 times)	47	7.0
	Often (3–4 times)	18	2.7
	Very often (5 or more times)	12	1.8
Suicidal threats	Never	626	93
	Once	38	5.6
	More than once	9	1.3
Likely hood of suicide in the future	Never	466	69.2
	No chance at all	77	11.4
	Rather unlikely	36	5.3
	Unlikely	36	5.3
	Likely	26	3.9
	Rather likely	27	4.0
	Very likely	5	0.7

Table 3 depicts that about 18.6% of the adolescents had suicidal behaviour. More than half (55.4%) of the adolescents had minor NSSI and less than one third (28.7%) of them had moderate to severe type of NSSI within past one year. On the other hand, more than two fifth (42.1%) adolescents had no any non -suicidal self–injury and more than half (57.9%) had NSSI in past one year. Less than two third (59.9%) of adolescents had performed Non-suicidal self–injury in their lifetime even if they had not done in past one year.

**Table 3:** Characteristics of adolescent’s behaviour in Pokhara Metropolitan, Nepal.

Variables (n = 673)	Category	Frequency	Percentage
Suicidal behaviour	No suicidal behaviour	548	81.4
	Suicidal behaviour	125	18.6
NSSI within past 1 year	Minor NSSI only	373	55.4
	Moderate/Severe NSSI only	193	28.7
	No NSSI	283	42.1
	NSSI	390	57.9
NSSI ever if not done in past one year	Yes	403	59.9
	No	270	40.1

Less than two fifth (38.2%) of the adolescents had picked at a wound followed by biting (24.2%), hitting on purpose (17.8%), pulling hair out (16.9%), scrapping skin (16.5%), cut or carved on skin (13.5%), inserting objects under nails or skin (13.1%). Other NSSI was only 0.6% that includes eating foreign objects, jumping from high place, pinching skin and closing mouth to stop breathing (Table 4).

**Table 4:** Non-suicidal self-injury activities among adolescents within past one year in Pokhara, Nepal.

Characteristics (n = 673)	Frequency	Percentage
picked at a wound	257	38.2
bit yourself (i.e. your mouth or lip)	163	24.2
hit yourself on purpose	120	17.8
pulled your hair out	114	16.9
scraped your skin	111	16.5
cut or carved on your skin	91	13.5
inserted objects under your nails or skin	88	13.1
erased your skin	53	6.5
picked areas of your body to the point of drawing blood	44	6.5
gave yourself a tattoo	43	6.4
burned your skin (i.e. with a cigarette, match or other hot objects)	24	3.6
Others	4	0.6

### Factors associated with suicidal behaviour among adolescents

In Model 2, multiple logistic regression analysis was performed without sleep problem, where females (AOR = 2.18, 95% CI = 1.44–3.29), adolescents from family having monthly income less than NPR 40,000 (AOR = 2.07, 95% CI = 1.24–3.46) and students from private schools (AOR = 2.60, 95% CI = 1.63–4.13) were more likely to have suicidal behaviour (Table 5).

**Table 5:** Factors associated with suicidal behaviour among adolescents in Pokhara, Nepal.

Variables	Model 1		Model 2		Model 3	
	UOR (95% CI)	*p-*value	AOR (95% CI)	*p-*value	AOR (95% CI)	*p-*value
**Age**						
Early adolescents (13–14)	1.77 (0.85–3.67)	0.124	1.44(0.66–3.12)	0.356	1.40(0.63–3.11)	0.398
Middle adolescents (15–17)	1.90 (1.02–3.55)	0.043	1.83(0.95–3.50)	0.068	1.87(0.97–3.60)	0.062
Late adolescents (18 and above)	Ref		Ref		Ref	
**Sex**						
Male	Ref		Ref		Ref	
Female	1.89 (1.28–2.81)	0.001	2.18 (1.44–3.29)	**<0.001** *	1.96 (1.28–3.00)	**0.002** *
**Ethnicity**						
Brahmin/Chhetri	0.96 (0.62–1.47)	0.853				
Janajati	0.70 (0.35–1.38)	0.304				
Other(Dalit, Madhesi, Muslim)	Ref					
**Monthly family income**						
NRS <40000 ($304.8657 USD)	1.37 (0.86–2.18)	0.176	2.07(1.24–3.46)	**0.005** *	1.97(1.16–3.35)	**0.011** *
NRS 40001–50000 ($304.8733–381.0821 USD)	0.91 (0.54–1.53)	0.739	1.18(0.68–2.05)	0.548	1.07(0.61–1.90)	0.794
NRS 50001 and above ($381.0897 USD and above)	Ref		Ref		Ref	
**Type of school**						
Private	1.90 (1.27–2.86)	0.002	2.60(1.63–4.13)	**<0.001** *	2.99 (1.84–4.86)	**<0.001** *
Public	Ref		Ref		Ref	
**Sleep problem**						
No	Ref		**-**	**-**	Ref	
Yes	3.60 (2.14–6.04)	<0.001	**-**	**-**	3.88 (2.27–6.63)	**<0.001** *

Statistically significant at p value <0.2 adjusted in Models

*Statistically significant at *p*<0.05 Model 2 R^2^ = 0.086, Model 3 R^2^ = 0.152.

Model 2 = adjusted for age, sex, monthly family income and type of school.

Model 3 = adjusted for age, sex, monthly family income, type of school and sleep problem.

UOR = Unadjusted Odds Ratio, AOR = Adjusted Odds Ratio.

In Model 3, sleep problem was added where female students (AOR = 1.96, 95% CI 1.28–3.00), adolescents from family having monthly income less than NPR 40,000 (AOR = 1.97 95% CI = 1.16–3.35) students from private schools (AOR = 2.99, CI = 1.84–4.86) and adolescents who had sleep problem (AOR = 3.88, 95% CI = 2.27–6.63) shows higher suicidal behaviour in comparison to their counterparts (Table 5).

After adjustment with all these variables with sleep problem in model 3, there was modification in the effect of explanatory variables on suicidal behaviour. Therefore, sleep problem plays an important role on exhibiting suicidal behaviour among adolescents. Further, Nagelkerke R^2^ value of 0.086 in model 2 explains that the 8.6% of the variation in the dependent variable (suicidal behavior) can be explained by the independent variables included in the analysis. Similarly, in model 3 Nagelkerke R^2^ value of 0.152 explains that the 15.2% of the variation in the dependent variable (suicidal behavior) can be explained by the explanatory variables included in the analysis. This concludes that after adding sleep problem in the analysis the variability also increases by 6.6% (Table 5).

### Factors associated with NSSI among adolescents

In Model 2, multiple logistic regression analysis was conducted excluding sleep problem which reveal that only students from private schools (AOR = 2.15, 95% CI 1.49–3.11) demonstrated a higher likelihood of engaging in NSSI. In Model 3, inclusion of sleep problem revealed that private school students (AOR = 2.52, 95% CI 1.71–3.72) and adolescents experiencing sleep problem (AOR = 3.24, 95% CI 2.26–4.65) were more susceptible to NSSI (Table 6).

**Table 6:** Factors associated with non-suicidal self-injury among adolescents in Pokhara, Nepal.

Variables	Model 1		Model 2		Model 3	
	UOR (95% CI)	*p-*value	AOR (95% CI)	*p*-value	AOR (95% CI)	*p-*value
**Age**						
Early adolescents (13–14)	1.76 (1.04–2.97)	0.033	1.31 (0.74–2.29)	0.344	1.37 (0.77–2.46)	0.279
Middle adolescents (15–17)	1.28 (0.84–1.95)	0.239	1.13 (0.73–1.76)	0.571	1.17 (0.74–1.83)	0.494
Late adolescents (18 and above)	Ref		Ref		Ref	
**Sex**						
Male	Ref					
Female	0.98 (0.72–1.33)	0.916				
**Ethnicity**						
Brahmin/Chhetri	0.98 (0.69–1.38)	0.924	0.87 (0.61–1.25)	0.463	1.09 (0.75–1.59)	0.63
Janajati	0.58 (0.35–0.96)	0.035	0.74 (0.44–1.26)	0.275	0.84 (0.49–1.44)	0.53
Other(Dalit, Madhesi, Muslim)	Ref		Ref		Ref	
**Monthly family income**						
NRS <40000 ($304.8657 USD)	0.80 (0.55–1.17)	0.260	1.21 (0.79–1.85)	0.367	1.15 (0.74–1.79)	0.524
NRS 40001–50000 ($304.8733–381.0821 USD)	0.52 (0.35–0.78)	0.001	0.71 (0.46–1.09)	0.127	0.64 (0.41–1.00)	0.051
NRS 50001 and above ($381.0897 USD and above)	Ref		Ref		Ref	
**Type of school**						
Private	2.30 (1.68–3.14)	<0.001	2.15 (1.49–3.11)	**<0.001** *	2.52 (1.71–3.72)	**<0.001** *
Public	Ref		Ref		Ref	
**Sleep problem**			-	-		
No	Ref		-	-	Ref	
Yes	2.53 (1.83–3.51)	<0.001	-	-	3.24 (2.26–4.65)	**<0.001** *

*Statistically significant at *p*<0.05 Model 2 R^2^ = 0.072, Model 3 R^2^ = 0.152.

Model 2 = adjusted for age, ethnicity, monthly family income and type of school.

Model 3 = adjusted for age, ethnicity, monthly family income, type of school and sleep problem.

UOR = Unadjusted Odds Ratio, AOR = Adjusted Odds Ratio.

After adjustment with all these variables with sleep problem in model 3, there was modification in the effect of explanatory variables on non -suicidal self -injury. Therefore, sleep problem plays an important role on exhibiting non-suicidal self -injury among adolescents. Further, Nagelkerke R^2^ value of 0.072 in model 2 explains that the 7.2% of the variation in the dependent variable (non -suicidal self -injury) can be explained by the independent variables included in the analysis. Similarly, in model 3 Nagelkerke R^2^ value of 0.152 explains that the 15.2% of the variation in the dependent variable (non -suicidal self–injury) can be explained by the explanatory variables included in the analysis. This concludes that after adding sleep problem in the analysis the variability also increases by 8% (Table 6).

## Discussion

In this study, sleep problem was present in 65.2% of adolescents, suicidal behaviour in 18.6% and non-suicidal self-injury in 57.9% of the adolescents. The main finding was that there was the association of sleep problem with suicidal behaviour and non-suicidal self-injury among adolescents even after adjustment with independent variables and variability also had increased after adding sleep problem. In addition to this findings, female gender and students from private schools were associated with suicidal behaviour. Similarly, adolescents from private schools were more likely to engage in NSSI.

### Sleep problem

The result of this study showed that 65.2% of adolescents experienced sleep problem, a figure similar to study from Saudi Arabia (65%), Kathmandu (59.1%), Turkey (58.6%) [48–50]. In Egypt 72.5% of adolescents were found to suffer from sleep problem. Other studies conducted in different places of Nepal reported rates of sleep problem ranging from31% to 39% [36,37,51]. Similar finding was reported in Finland where comparable prevalence was noted [52]. In Croatia, a prominently high proportion of adolescents (86.7%) reported experiencing sleep problems [53]. Furthermore, in US 80% students were found to be affected by sleep problem [54].

### Suicidal behaviour

One of the leading causes of death among teenagers worldwide is suicide [55]. In this study, 18.6% of adolescents had suicidal behaviour in their lifetime. Similarly, 18.4% reported experiencing suicidal ideation which was almost consistent with Mongolian students (19.8%) and Chinese adolescents (16.6%) but contrasting with rates among Taiwan (12.6%) American (34.7%) and Bhutanese adolescents (11.6%) [26,28,56–59]. However, Global School Based Health Survey (GSBHS) in Nepal reported lower prevalence of suicidal ideation (13.59%) as compared to finding of present study [39]. This reflects that the suicidal behavior is in the increasing trend in Nepal.

In Bangladesh, university students (aged 18 and above) reported a prevalence of 12.8% for suicidal ideation [60]. Likewise, suicidal plan was reported by 6.5% of adolescents in this study. Our finding was corresponds with observation made in China (9.6%) [56]. However it was not consistent with findings among Mongolian students (12.8%) [26].

A GSBHS in Bhutan reported 11.3% prevalence of suicidal attempt [58]. American adolescents showed 14.9% of suicidal attempt in a study where participants were selected from Collaborative Adolescent Research on Suicide and Emotions (CARES) trial [59]. The variation in prevalence rates of suicidal behaviour among different countries may be attributable to the effects of numerous risk factors. This variations is likely due to the existence of numerous underlying, complex, and interconnected components of suicidal behavior at the individual, community, and society levels [58].

In this study, females were 1.96 times more likely to exhibit suicidal behaviour (AOR = 1.96, 95% CI = 1.28–3.00). Similar finding was reported, supported by studies conducted in China, United States and Mongolia [13,26,61]. A Global School Based Student Health Survey in Bhutan also reported that female students were more likely to display suicidal behaviour [58]. It is believed that these biological and socially constructed differences contribute to this gender association [58].

Adolescents from private schools were 2.99 times more likely to report suicidal behaviour (AOR = 2.99, 95%CI = 1.84–4.86) in comparison to their public school counterparts in this study. The presence of academic stress and heightened pressure among adolescents attending private schools reported from Nepal and India [14,15]. Similarly, adolescents residing in private school hostel reported heightened level of perceived stress [62]. Consequently, future studies should focus on academic stress, as failure to address this issue in a timely manner may elevate the risk of suicidal behaviour.

Moreover, adolescents those experience sleep problem are 3.88 times more likely to be engaged in suicidal behaviour (AOR = 3.88, 95% CI = 2.27–6.63). A systematic review and meta-analysis found a significant association between sleep problems and suicidal behavior [25]. Similarly, a study by Wojnar et.al. reported significant association between sleep problem and suicidal behavior [4]. To support this, Bhutan Global School Based Student Health Survey reported the similar finding [58]. Additionally, a longitudinal study revealed that experiencing sleep problems at ages 12 to 14 strongly predicted suicidal thoughts and self-harm behaviors at ages 15 to 17, even after adjusting for gender, parental drinking, earlier suicidal thoughts or self-harm behaviors at ages 12 to 14 [31]. The underlying cause could be that not getting enough sleep affects cognitive function, which can lead to poor judgment, problems with impulse control, increased exhaustion, and feelings of hopelessness, all of which can contribute to suicidal thoughts and behavior [28,63].

### Non-suicidal self-injury

NSSI is a frequently encountered but often concealed activity, particularly among adolescents. The prevalence of NSSI was reported 57.9% among adolescents in this study. This proportion is consistent with other studies from Spain (55.6%), US (55%) and China (51.40%) [17,64]. However, Brazil (45.3%), Sweden (41.6%) South Korea (28.3%), Portugal (20.3%) had different findings [65–68]. The disparity in prevalence rates may be based on by variations in the sample’s cultural background as well as the use of various techniques to evaluate self-injurious behavior. The way in which NSSI was measured, an anonymous self-report on a comprehensive list of numerous self-harm behaviors, could be one explanation for our findings. NSSI evaluation varies greatly, and it’s probable that more thorough, widely defined assessments that include cued listings of self-harm behaviors [69], as opposed to free-response survey formats, catch a wider range of NSSI and hence produce a higher rate of NSSI [17,70].

Picking at wounds, self-biting, hitting oneself on purpose and scrapping skin were the most commonly reported NSSI behaviors. However, some research suggests that biting oneself is a socially accepted and normal habit [64] and that picking at the wound is a clinically of little importance behavior [17].

Moreover, students from private schools were 2.52 times more likely to be engaged in NSSI (AOR = 2.52 95%CI 1.71–3.72) in Nepal. High prevalence of NSSI among private school students than public school was reported by Poudel et.al. in same setting [18]. The possible reason could be that private schools typically have longer teaching hours and impose greater academic pressure compared to public schools, potentially influencing adolescents to engage in NSSI. In addition, adolescents having sleep problem were 3.24 times more likely to show NSSI (AOR = 3.24, 95%CI 2.26–4.65). Lack of sleep may enhance emotional distress while impairing cognitive abilities (such as concentration, memory, judgment, and problem-solving) necessary for an effective way to cope with emotional pain. Most experts acknowledge that NSSI’s primary purpose is to control emotional distress [71,72]. It follows that poor sleep may increase the risk for NSSI if it is assumed that NSSI is a less cognitively demanding form of emotion regulation (i.e., requires less of concentration, memory, judgment, and problem-solving than many other forms of emotion regulation) and that poor sleep generally increases the likelihood that the person turns to less cognitively demanding forms of emotion regulation [71].

The main objective of this study was to assess the role of sleep problem on suicidal behaviour and non-suicidal self-injury among adolescents which was further clarified by adjusted relationship between the variables in Tables 5 and 6. Regarding relationship between sleep problem and suicidal behaviour, model 2 explains that the 8.6% of the variation in the suicidal behavior can be explained by the independent variables included in the analysis. Similarly, in model 3, 15.2% of the variation in suicidal behavior can be explained by the explanatory variables included in the analysis. This concludes that after adding sleep problem in the analysis the variability also increases by 6.6%. One study from Switzerland used multivariate analysis, and it found that sleep issues significantly increased the variation in suicide attempts [73].

Regarding relationship between sleep problem and non-suicidal self-injury, model 2 reported that 7.2% of the variation in non-suicidal self-injury which was explained by the independent variables included in the analysis. Similarly, in model 3, 15.2% of the variation in non-suicidal self-injury was explained by the explanatory variables included in the analysis. This concludes that after adding sleep problem in the analysis the variability also increases by 8% which somehow proves that sleep problem plays an important role in having non -suicidal self-injury.

### Policy and program implications

The public health program focused on school children in the context of Nepal was solely the school health program, which didn’t cover the issue of adolescent mental health. Despite the high prevalence of behavioral and mental issues reported among school students, there are no mental health-related program designed specifically for adolescent children. The increasing use of mobile phone and internet addiction among adolescent and its consequences on mental health, are observable in the Nepalese context [51]. This study confirms the link of sleep problem with suicidal behavior and non-suicidal self-injury, so the school curriculum should incorporate the sleep-related issues along with mental health education.

The policy should design a facility for a regular monitoring system of mental health among adolescent in school setting. The initial step could be implemented in private school settings, focusing on female adolescents and screening for sleep problems should be emphasized.

### Limitations

The limitation of this study is that the results of the study depend on how adolescents responded to a self-administered questionnaire and not verified by other source, which could lead to recalling bias for assessing sleep problem, NSSI, suicidal behaviour and refusal to reveal personal information, which could have resulted in either over reporting or underreporting of information. Although electroencephalography and actigraphy are ideal objective measurements of sleep duration and sleep difficulties, self-reports and interviews continue to be the preferred methods in large-scale epidemiologic investigations.

Due to the stigma associated with Suicidal behaviour in culturally diverse Nepalese civilizations, the symptoms may have gone unreported, suggesting that there is social desirability bias. The adolescent who did not attend school is not included in the study. Because of the cross-sectional nature of this study, it is impossible to establish a causal link between sleep issues, NSSI, and suicidal behaviour. This could be evaluated only with carefully planned, prospective follow-up studies.

## Conclusion

This study concludes that nearly two third of adolescents experienced sleep problem, nearly one fifth exhibited suicidal behavior and more than a half exhibited NSSI. Sleep problem had significant influence on suicidal behavior and non-suicidal self-injury, increasing the risk for both by less than one tenth. These findings highlighted the urgent need for targeted screening and support mechanisms tailored to address adolescent sleep and suicidal behavior. Failing to address these issue promptly could have profound long-term implications for adolescent mental health and wellbeing.

## Supporting information

S1 Dataset (SAV)

## References

[1] Léger D, Poursain B, Neubauer D, Uchiyama M. An international survey of sleeping problems in the general population. Curr Med Res Opin. 2008;24: 307–317. doi: 10.1185/030079907x253771 18070379

[2] Stranges S, Tigbe W, Gómez-Olivé FX, Thorogood M, Kandala N-B. Sleep Problems: An Emerging Global Epidemic? Findings From the INDEPTH WHO-SAGE Study Among More Than 40,000 Older Adults From 8 Countries Across Africa and Asia. Sleep. 2012;35: 1173–1181. doi: 10.5665/sleep.2012 22851813 PMC3397790

[3] Koyanagi A, Stickley A. The Association between Sleep Problems and Psychotic Symptoms in the General Population: A Global Perspective. Sleep. 2015;38: 1875–1885. doi: 10.5665/sleep.5232 26085291 PMC4667394

[4] Wojnar M, Ilgen MA, Wojnar J, McCammon RJ, Valenstein M, Brower KJ. Sleep Problems and Suicidality in the National Comorbidity Survey Replication. J Psychiatr Res. 2009;43: 526–531. doi: 10.1016/j.jpsychires.2008.07.006 18778837 PMC2728888

[5] Paruthi S, Brooks LJ, D’Ambrosio C, Hall WA, Kotagal S, Lloyd RM, et al. Consensus Statement of the American Academy of Sleep Medicine on the Recommended Amount of Sleep for Healthy Children: Methodology and Discussion. J Clin Sleep Med. 2016;12: 1549–1561. doi: 10.5664/jcsm.6288 27707447 PMC5078711

[6] Rasekhi S, Pour Ashouri F, Pirouzan A. Effects of Sleep Quality on the Academic Performance of Undergraduate Medical Students. Health Scope. 2016;5. doi: 10.17795/jhealthscope-31641

[7] Ismail DM, Mahran DG, Zarzour AH, Sheahata GA. Sleep Quality and Its Health Correlates Among Egyptian Secondary School Students. J Soc Behav Health Sci. 2017;11. doi: 10.5590/JSBHS.2017.11.1.5

[8] Bøe T, Hysing M, Stormark KM, Lundervold AJ, Sivertsen B. Sleep problems as a mediator of the association between parental education levels, perceived family economy and poor mental health in children. J Psychosom Res. 2012;73: 430–436. doi: 10.1016/j.jpsychores.2012.09.008 23148810

[9] Nock MK, Borges G, Bromet EJ, Cha CB, Kessler RC, Lee S. Suicide and Suicidal Behavior. Epidemiol Rev. 2008;30: 133–154. doi: 10.1093/epirev/mxn002 18653727 PMC2576496

[10] Abio A, Owusu PN, Posti JP, Bärnighausen T, Shaikh MA, Shankar V, et al. Cross-national examination of adolescent suicidal behavior: a pooled and multi-level analysis of 193,484 students from 53 LMIC countries. Soc Psychiatry Psychiatr Epidemiol. 2022;57: 1603–1613. doi: 10.1007/s00127-022-02287-x 35445842 PMC9288956

[11] WHO. Mental Health Status of Adolescents in South-East Asia: Evidence for Action. New Delhi, India: WHO, South East Asia; 2017 Apr. Available: https://apps.who.int/iris/handle/10665/254982.

[12] Dangi J, Dangi J, Thagunna N, Khayamali R, Subba usha kiran. Suicidal Ideation among Nepalese Adolescents. Mind Soc. 2022;11: 33–40. doi: 10.56011/mind-mri-112-20223

[13] Liu X, Tein J-Y, Zhao Z, Sandler IN. Suicidality and correlates among rural adolescents of China. J Adolesc Health. 2005;37: 443–451. doi: 10.1016/j.jadohealth.2004.08.027 16310121

[14] Gurung M, Chansatitporn N, Chamroonsawasdi K, Lapvongwatana P. Academic Stress among High School Students in a Rural Area of Nepal: A Descriptive Cross-sectional Study. J Nepal Med Assoc. 2020;58: 306–309. doi: 10.31729/jnma.4978 32538923 PMC7654462

[15] Deb S, Strodl E, Sun J. Academic-related stress among private secondary school students in India. Asian Educ Dev Stud. 2014;3. doi: 10.1108/AEDS-02-2013-0007

[16] Kim SH, Kim J-S, Yoo HY, Ryu E. Parental Occupational Status and Suicidal Ideation in Adolescent: Cross-Sectional Secondary Data Analysis. J Pediatr Nurs. 2019;45: e57–e63. doi: 10.1016/j.pedn.2019.01.005 30670322

[17] Lloyd-Richardson EE, Perrine N, Dierker L, Kelley ML. Characteristics and functions of non-suicidal self-injury in a community sample of adolescents. Psychol Med. 2007;37: 1183–1192. doi: 10.1017/S003329170700027X 17349105 PMC2538378

[18] Poudel A, Lamichhane A, Magar KR, Khanal GP. Non suicidal self injury and suicidal behavior among adolescents: co-occurrence and associated risk factors. BMC Psychiatry. 2022;22: 96. doi: 10.1186/s12888-022-03763-z 35139825 PMC8827284

[19] Lim K-S, Wong CH, McIntyre RS, Wang J, Zhang Z, Tran BX, et al. Global Lifetime and 12-Month Prevalence of Suicidal Behavior, Deliberate Self-Harm and Non-Suicidal Self-Injury in Children and Adolescents between 1989 and 2018: A Meta-Analysis. Int J Environ Res Public Health. 2019;16: 4581. doi: 10.3390/ijerph16224581 31752375 PMC6888476

[20] De Luca L, Pastore M, Palladino BE, Reime B, Warth P, Menesini E. The development of Non-Suicidal Self-Injury (NSSI) during adolescence: A systematic review and Bayesian meta-analysis. J Affect Disord. 2023;339: 648–659. doi: 10.1016/j.jad.2023.07.091 37479039

[21] Hamdan S, Apter A, Levi-Belz Y. Non-suicidal Self-Injury Among Adolescents From Diverse Ethnocultural Groups in Israel: The Association With Sleep Problems and Internet Addiction. Front Psychiatry. 2022;13. Available: https://www.frontiersin.org/articles/10.3389/fpsyt.2022.899956. doi: 10.3389/fpsyt.2022.899956 35633814 PMC9136052

[22] Fan Y, Liu J, Zeng Y, Conrad R, Tang Y. Factors Associated With Non-suicidal Self-Injury in Chinese Adolescents: A Meta-Analysis. Front Psychiatry. 2021;12: 747031. doi: 10.3389/fpsyt.2021.747031 34916971 PMC8669619

[23] Lodebo BT, Möller J, Larsson J-O, Engström K. Socioeconomic position and self-harm among adolescents: a population-based cohort study in Stockholm, Sweden. Child Adolesc Psychiatry Ment Health. 2017;11: 46. doi: 10.1186/s13034-017-0184-1 28878818 PMC5585967

[24] McGlinchey EL, Courtney-Seidler EA, German M, Miller AL. The Role of Sleep Disturbance in Suicidal and Nonsuicidal Self-Injurious Behavior among Adolescents. Suicide Life Threat Behav. 2017;47: 103–111. doi: 10.1111/sltb.12268 27273654

[25] Liu J-W, Tu Y-K, Lai Y-F, Lee H-C, Tsai P-S, Chen T-J, et al. Associations between sleep disturbances and suicidal ideation, plans, and attempts in adolescents: a systematic review and meta-analysis. Sleep. 2019;42: zsz054. doi: 10.1093/sleep/zsz054 30843072

[26] Altangerel U, Liou J-C, Yeh P-M. Prevalence and Predictors of Suicidal Behavior Among Mongolian High School Students. Community Ment He alth J. 2014;50: 362–372. doi: 10.1007/s10597-013-9657-8 24282032

[27] Blank M, Zhang J, Lamers F, Taylor AD, Hickie IB, Merikangas KR. Health Correlates of Insomnia Symptoms and Comorbid Mental Disorders in a Nationally Representative Sample of US Adolescents. Sleep. 2015;38: 197–204. doi: 10.5665/sleep.4396 25325502 PMC4288600

[28] Liu X. Sleep and Adolescent Suicidal Behavior. SLEEP. 2018;27: 1351–8. 10.1093/sleep/27.7.1351 15586788

[29] Wang X, Cheng S, Xu H. Systematic review and meta-analysis of the relationship between sleep disorders and suicidal behaviour in patients with depression. BMC Psychiatry. 2019;19: 303. doi: 10.1186/s12888-019-2302-5 31623600 PMC6798511

[30] Matamura M, Tochigi M, Usami S, Yonehara H, Fukushima M, Nishida A, et al. Associations between sleep habits and mental health status and suicidality in a longitudinal survey of monozygotic twin adolescents. J Sleep Res. 2014;23: 292–296. doi: 10.1111/jsr.12127 24456111

[31] Wong MM, Brower KJ, Zucker RA. Sleep problems, suicidal ideation, and self-harm behaviors in adolescence. J Psychiatr Res. 2011;45: 505–511. doi: 10.1016/j.jpsychires.2010.09.005 20889165 PMC3026917

[32] Khazaie H, Zakiei A, McCall WV, Noori K, Rostampour M, Sadeghi Bahmani D, et al. Relationship between Sleep Problems and Self-Injury: A Systematic Review. Behav Sleep Med. 2021;19: 689–704. doi: 10.1080/15402002.2020.1822360 32991212

[33] Latina D, Bauducco S, Tilton-Weaver L. Insomnia symptoms and non-suicidal self-injury in adolescence: understanding temporal relations and mechanisms. J Sleep Res. 2021;30: e13190. doi: 10.1111/jsr.13190 32893426 PMC7900995

[34] Wang J-N, Liu L, Wang L. Prevalence and associated factors of emotional and behavioural problems in Chinese school adolescents: a cross-sectional survey. Child Care Health Dev. 2014;40: 319–326. doi: 10.1111/cch.12101 23952583

[35] Bøe T, Hysing M, Skogen JC, Breivik K. The Strengths and Difficulties Questionnaire (SDQ): Factor Structure and Gender Equivalence in Norwegian Adolescents. PloS One. 2016;11: e0152202. doi: 10.1371/journal.pone.0152202 27138259 PMC4854391

[36] Karki K, Singh DR, Maharjan D, K. C. S, Shrestha S, Thapa DK. Internet addiction and sleep quality among adolescents in a peri-urban setting in Nepal: A cross-sectional school-based survey. Veauthier C, editor. PLOS ONE. 2021;16: e0246940. doi: 10.1371/journal.pone.0246940 33600410 PMC7891762

[37] Gautam P, Dahal M, Baral K, Acharya R, Khanal S, Kasaju A, et al. Sleep Quality and Its Correlates among Adolescents of Western Nepal: A Population-Based Study. Pillar G, editor. Sleep Disord. 2021;2021: 1–8. doi: 10.1155/2021/5590715 34055416 PMC8143896

[38] Singh DR, Sunuwar DR, Dahal B, Sah RK. The association of sleep problem, dietary habits and physical activity with weight status of adolescents in Nepal. BMC Public Health. 2021;21: 938. doi: 10.1186/s12889-021-10985-5 34001092 PMC8130305

[39] Pandey AR, Bista B, Dhungana RR, Aryal KK, Chalise B, Dhimal M. Factors associated with suicidal ideation and suicidal attempts among adolescent students in Nepal: Findings from Global School-based Students Health Survey. PLOS ONE. 2019;14: e0210383. doi: 10.1371/journal.pone.0210383 31002715 PMC6474648

[40] Cipriano A, Cella S, Cotrufo P. Nonsuicidal Self-injury: A Systematic Review. Front Psychol. 2017;8. Available: https://www.frontiersin.org/articles/10.3389/fpsyg.2017.01946. doi: 10.3389/fpsyg.2017.01946 29167651 PMC5682335

[41] Government of Nepal. National Population and Housing Census 2021. Thapathali, Kathmandu, Nepal: National Statistics Office; 2023 Mar. Available: https://censusnepal.cbs.gov.np/results/files/result-folder/National%20Report_English.pdf.

[42] Oka Y Y, Horiuchi F, Tanigawa T, Suzuki S, Kondo F, Sakurai S. Development of a new sleep screening questionnaire: Child and Adolescent Sleep Checklist (CASC). Japanese Journal of Sleep Medicine. 2009;3: 404–408.

[43] Sivertsen B, Overland S, Glozier N, Bjorvatn B, Maeland JG, Mykletun A. The effect of OSAS on sick leave and work disability. Eur Respir J. 2008;32: 1497–1503. doi: 10.1183/09031936.00044908 18653651

[44] Sivertsen B, Pallesen S, Sand L, Hysing M. Sleep and body mass index in adolescence: results from a large population-based study of Norwegian adolescents aged 16 to 19 years. BMC Pediatr. 2014;14: 204. doi: 10.1186/1471-2431-14-204 25128481 PMC4148405

[45] Lloyd EE. Self-Mutilation in a Community Sample of Adolescents. 1997. Available: https://digitalcommons.lsu.edu/gradschool_disstheses/6546.

[46] Nock MK, Prinstein MJ, Sterba SK. Revealing the form and function of self-injurious thoughts and behaviors: A real-time ecological assessment study among adolescents and young adults. J Abnorm Psychol. 2009;118: 816–827. doi: 10.1037/a0016948 19899851 PMC5258190

[47] Osman A, Bagge CL, Guitierrez PM, Konick LC, Kopper BA, Barrios FX. The Suicidal Behaviors QuestionnaireRevised (SBQ-R): Validation with clinical and nonclinical samples, Assessment. 2001;8: 443–454. doi: 10.1177/107319110100800409 11785588

[48] Merdad RA, Merdad LA, Nassif RA, El-Derwi D, Wali SO. Sleep habits in adolescents of Saudi Arabia; distinct patterns and extreme sleep schedules. Sleep Med. 2014;15: 1370–1378. doi: 10.1016/j.sleep.2014.06.008 25260431

[49] Koças F, Şaşmaz T. Internet addiction increases poor sleep quality among high school students. Turk J Public Health. 2018;16: 11.

[50] Khadka R, Bista S, Baskota S, Poudel L, Gurung M. Sleep Quality among College Students in Kathmandu Valley, Nepal. Nepal Med J. 2019;2. doi: 10.37080/nmj.61

[51] Bhandari PM, Neupane D, Rijal S, Thapa K, Mishra SR, Poudyal AK. Sleep quality, internet addiction and depressive symptoms among undergraduate students in Nepal. BMC Psychiatry. 2017;17: 106. doi: 10.1186/s12888-017-1275-5 28327098 PMC5361804

[52] Tynjala J. Perceived sleep quality and its precursors in adolescents. Health Promot Int. 1999;14: 155–166. doi: 10.1093/heapro/14.2.155

[53] Franić T, Kralj Ž, Marčinko D, Knez R, Kardum G. Suicidal ideations and sleep-related problems in early adolescence: Suicidal ideations and sleep-related problems. Early Interv Psychiatry. 2014;8: 155–162. doi: 10.1111/eip.12035 23445244

[54] Megdal SP, Schernhammer ES. Correlates for poor sleepers in a Los Angeles high school. Sleep Med. 2007;9: 60–63. doi: 10.1016/j.sleep.2007.01.012 17869576

[55] Redmore J, Kipping R, Trickey A, May MT, Gunnell D. Analysis of trends in adolescent suicides and accidental deaths in England and Wales, 1972–2011. Br J Psychiatry. 2016;209: 327–333. doi: 10.1192/bjp.bp.114.162347 27284083 PMC5046738

[56] Chen J, Wan Y, Sun Y, Tao F. [Relations between problems on sleeping and suicidal behaviors in middle school students]. Zhonghua Liu Xing Bing Xue Za Zhi Zhonghua Liuxingbingxue Zazhi. 2014;35: 129–133. 24739549

[57] Isaac V, Wu C-Y, McLachlan CS, Lee M-B. Associations between health-related self-efficacy and suicidality. BMC Psychiatry. 2018;18: 126. doi: 10.1186/s12888-018-1705-z 29747578 PMC5946427

[58] Dema T, Tripathy JP, Thinley S, Rani M, Dhendup T, Laxmeshwar C, et al. Suicidal ideation and attempt among school going adolescents in Bhutan–a secondary analysis of a global school-based student health survey in Bhutan 2016. BMC Public Health. 2019;19: 1605. doi: 10.1186/s12889-019-7791-0 31791280 PMC6889681

[59] Asarnow JR, Bai S, Babeva KN, Adrian M, Berk MS, Asarnow LD, et al. Sleep in youth with repeated self‐harm and high suicidality: Does sleep predict self‐harm risk? Suicide Life Threat Behav. 2020;50: 1189–1197. doi: 10.1111/sltb.12658 32706147 PMC9327783

[60] Tasnim R, Islam MdS, Sujan MdSH, Sikder MdT, Potenza MN. Suicidal ideation among Bangladeshi university students early during the COVID-19 pandemic: Prevalence estimates and correlates. Child Youth Serv Rev. 2020;119: 105703. doi: 10.1016/j.childyouth.2020.105703 33204046 PMC7654299

[61] Kim YJ, Moon SS, Kim MJ. Physical and Psycho-Social Predictors of Adolescents’ Suicide Behaviors. Child Adolesc Soc Work J. 2011;28: 421–438. doi: 10.1007/s10560-011-0241-1

[62] Shrestha R, Timalsina S, Shakya R, Shrestha N, Kotera Y, Hashemy T, et al. Stress and Coping Mechanism among Students Residing in Private School Hostels. Ment Illn. 2023;2023: e6535583. doi: 10.1155/2023/6535583

[63] Goldstein TR, Bridge JA, Brent DA. Sleep disturbance preceding completed suicide in adolescents. J Consult Clin Psychol. 2008;76: 84–91. doi: 10.1037/0022-006X.76.1.84 18229986 PMC2823295

[64] Calvete E, Orue I, Brotherton H. Prevalence and functions of non-suicidal self-injury in Spanish adolescents. Psicothema. 2015; 223–228. doi: 10.7334/psicothema2014.262 26260928

[65] Zetterqvist M, Lundh L-G, Dahlström Ö, Svedin CG. Prevalence and Function of Non-Suicidal Self-Injury (NSSI) in a Community Sample of Adolescents, Using Suggested DSM-5 Criteria for a Potential NSSI Disorder. J Abnorm Child Psychol. 2013;41: 759–773. doi: 10.1007/s10802-013-9712-5 23344701

[66] Gaspar S, Reis M, Sampaio D, Guerreiro D, De Matos MG. Non-suicidal Self-Injuries and Adolescents High Risk Behaviours: Highlights from the Portuguese HBSC Study. Child Indic Res. 2019;12: 2137–2149. doi: 10.1007/s12187-019-09630-w

[67] Costa RPDO Peixoto ALRP, Lucas CCA Falcão DN, Farias JTDS Viana LFP, et al. Profile of non-suicidal self-injury in adolescents: interface with impulsiveness and loneliness. J Pediatr (Rio J). 2021;97: 184–190. doi: 10.1016/j.jped.2020.01.006 32151605 PMC9432040

[68] Lee J, Kim H, Kim S, Kim J, Shin I, Kim S. Non‐suicidal self‐injury is associated with psychotic like experiences, depression, and bullying in Korean adolescents. Early Interv Psychiatry. 2021;15: 1696–1704. doi: 10.1111/eip.13115 33461244

[69] Zoroglu SS, Tuzun U, Sar V, Tutkun H, Savacs HA, Ozturk M, et al. Suicide attempt and self-mutilation among Turkish high school students in relation with abuse, neglect and dissociation. Psychiatry Clin Neurosci. 2003;57: 119–126. doi: 10.1046/j.1440-1819.2003.01088.x 12519464

[70] Muehlenkamp JJ, Gutierrez PM. An Investigation of Differences Between Self-Injurious Behavior and Suicide Attempts in a Sample of Adolescents. Suicide Life Threat Behav. 2004;34: 12–23. doi: 10.1521/suli.34.1.12.27769 15106884

[71] Klonsky ED. The functions of deliberate self-injury: A review of the evidence. Clin Psychol Rev. 2007;27: 226–239. doi: 10.1016/j.cpr.2006.08.002 17014942

[72] Klonsky ED. The functions of self-injury in young adults who cut themselves: Clarifying the evidence for affect-regulation. Psychiatry Res. 2009;166: 260–268. doi: 10.1016/j.psychres.2008.02.008 19275962 PMC2723954

[73] Gex CR, Narring F, Ferron C, Michaud P-A. Suicide attempts among adolescents in Switzerland: prevalence, associated factors and comorbidity. Acta Psychiatr Scand. 1998;98: 28–33. doi: 10.1111/j.1600-0447.1998.tb10038.x 9696511

